# Integrative Multi-Omics Analysis of Oncogenic EZH2 Mutants: From Epigenetic Reprogramming to Molecular Signatures

**DOI:** 10.3390/ijms241411378

**Published:** 2023-07-12

**Authors:** Julian Aldana, Miranda L. Gardner, Michael A. Freitas

**Affiliations:** 1 Ohio State Biochemistry Program, Department of Chemistry and Biochemistry, The Ohio State University, Columbus, OH 43210, USA; aldanaaroca.1@buckeyemail.osu.edu (J.A.); gardner.207@osu.edu (M.L.G.); 2 Department of Cancer Biology and Genetics, Wexner Medical Center, The Ohio State University, Columbus, OH 43210, USA

**Keywords:** multi-omics, EZH2, epigenetics, mutations, histone methylation

## Abstract

Somatic heterozygous mutations in the active site of the enhancer of zeste homolog 2 (EZH2) are prevalent in diffuse large B-cell lymphoma (DLBCL) and acute myeloid leukemia (AML). The methyltransferase activity of EZH2 towards lysine 27 on histone H3 (H3K27) and non-histone proteins is dysregulated by the presence of gain-of-function (GOF) and loss-of-function (LOF) mutations altering chromatin compaction, protein complex recruitment, and transcriptional regulation. In this study, a comprehensive multi-omics approach was carried out to characterize the effects of differential H3K27me3 deposition driven by EZH2 mutations. Three stable isogenic mutants (EZH2^Y641F^, EZH2^A677G^, and EZH2H^689A/F667I^) were examined using EpiProfile, H3K27me3 CUT&Tag, ATAC-Seq, transcriptomics, label-free proteomics, and untargeted metabolomics. A discrete set of genes and downstream targets were identified for the EZH2 GOF and LOF mutants that impacted pathways involved in cellular proliferation, differentiation, and migration. Disruption of protein networks and metabolic signatures able to sustain aberrant cell behavior was observed in response to EZH2 mutations. This systems biology-based analysis sheds light on EZH2-mediated cell transformative processes, from the epigenetic to the phenotypic level. These studies provide novel insights into aberrant EZH2 function along with targets that can be explored for improved diagnostics/treatment in hematologic malignancies with mutated EZH2.

## 1. Introduction

The enhancer of zeste homolog 2 (EZH2) is a histone methyltransferase involved in epigenetic control of gene expression with a major role in hematopoietic stem cell differentiation, self-renewal, and lineage commitment [1,2]. It specifically catalyzes the sequential mono-(me), di-(me2), and tri-(me3) methylation of lysine residue 27 on histone H3 variants (H3K27) in an s-adenosyl methionine (SAM)-dependent reaction [3,4,5]. Lysine methylation stabilizes positive charge on histone tails and strengthens histone–DNA interaction, leading to a reduction in chromatin accessibility [6]. Therefore, H3K27me3 is considered a repressing epigenetic mark and EZH2 is associated with transcriptional downregulation via chromatin compaction and recruitment of other chromatin modifiers [7]. EZH2 is also able to display methyltransferase activity on non-histone proteins, including several transcription factors, and can also act as a co-activator depending on the cellular context [8].

EZH2 activity is tightly regulated by several cellular mechanisms throughout the cell cycle and developmental stages [9]. A multi-protein complex known as polycomb repressive complex 2 (PRC2) controls EZH2 recruitment, specificity, and activity [6,10]. EZH2 associates with other PRC2 members, including SUZ12, EED, RBAP46/48, and AEBP2 [4,5]. The PRC2 complex directs EZH2 to target genes through interactions with transcription factors (e.g., YY1 [11], SNAIL [12], and MYC [13]) and long non-coding RNAs (e.g., *HOTAIR* [14,15], *ANRIL* [16,17], and *MALAT1* [18,19,20]). EZH2 is further subject to post-translational modifications, including acetylation, phosphorylation [21,22], methylation, and ubiquitination that modulate its function and binding partners. Interestingly, it has been suggested that co-substrate availability exerts control over EZH2 histone methylation by limiting the amounts of SAM via central carbon metabolism and the methionine salvage pathway [23].

Heterozygous mutations of EZH2, particularly in its catalytic domain, are commonly found in hematologic malignancies [1,3]. Gain-of-function (GOF) mutations in Y641 and A677 are highly prevalent (frequency > 20%) in germinal center B-cell diffuse large B-cell lymphoma (GCB DLBCL) and follicular lymphoma (FL) [24,25,26], whereas loss-of-function (LOF) mutations located in F667, H689, and Y726 characterize myelodysplastic syndrome (MDS) and acute myeloid leukemia (AML) [27,28,29]. General population studies on EZH2 mutational status have not been yet conducted. Reported studies have been limited to lymphoma and leukemia patients to assess EZH2 mutation frequency. However, Morin et al. analyzed benign samples, finding 0% frequency in EZH2 mutations, versus 21% in GCB DLBCL [25]. Given the higher prevalence of DLBCL in adult patients, mutational status of EZH2 appear to influence adults the most [30,31]. Therefore, it has been suggested that EZH2 mutations are result of genetic instability in blood malignances.

The resulting dysregulation driven by EZH2 mutations has shown contrasting impacts in clinical cohorts, acting either as an oncogene or tumor suppressor. For instance, increased EZH2 activity often results in an enrichment of H3K27me3 levels, preferentially located around canonical PRC2 target genes for transcriptional silencing [32,33,34], which promotes lymphomagenesis by restraining plasmacytic differentiation and facilitating B-cell proliferation [26]. However, several reports have shown that EZH2 can activate transcription in a non-canonical fashion [21,22,35]. In the last decade, significant efforts have been devoted to understanding EZH2 mutant-driven activity to develop enhanced therapies towards EZH2 [36,37] and downstream effectors. Known PRC2 targets in hematopoietic stem cells (HSCs) include genes related to cell proliferation (*CDKN1A*, *CDKN2A* [38]), differentiation (*IDA* [38], *SOX7* [39]), and apoptosis (*NOXA*, *p21*, and *WIG1* [38]). Additional canonical/non-canonical targets and downstream cellular effects altered by abnormal EZH2 activity are still not completely understood.

In this study, the role of EZH2 GOF and LOF mutations in isogenic cells was interrogated under a comprehensive multi-omics approach by integrating global histone PTM analysis via EpiProfile, with mapping of H3K27me3 deposition across the genome using Cleavage Under Targets and Tagmentation (CUT&Tag), chromatin accessibility via Assay for Transposase-Accessible Chromatin with sequencing (ATAC-Seq), RNA-Seq, label-free bottom-up proteomics, and an MS-based untargeted metabolomics assay. An extensive multi-omics overview of how EZH2 mutations alter cellular epigenetics, gene expression and downstream protein and metabolite levels is provided herein. This study enhances our understanding of EZH2′s roles in mutant driven cancer and contributes a stronger molecular basis for developing and improving therapeutic interventions in EZH2 driven blood malignancies.

## 2. Results and Discussion

### 2.1. EZH2 Point Mutations Reshape the Cellular Epigenetic Landscape

**Figure 1:**
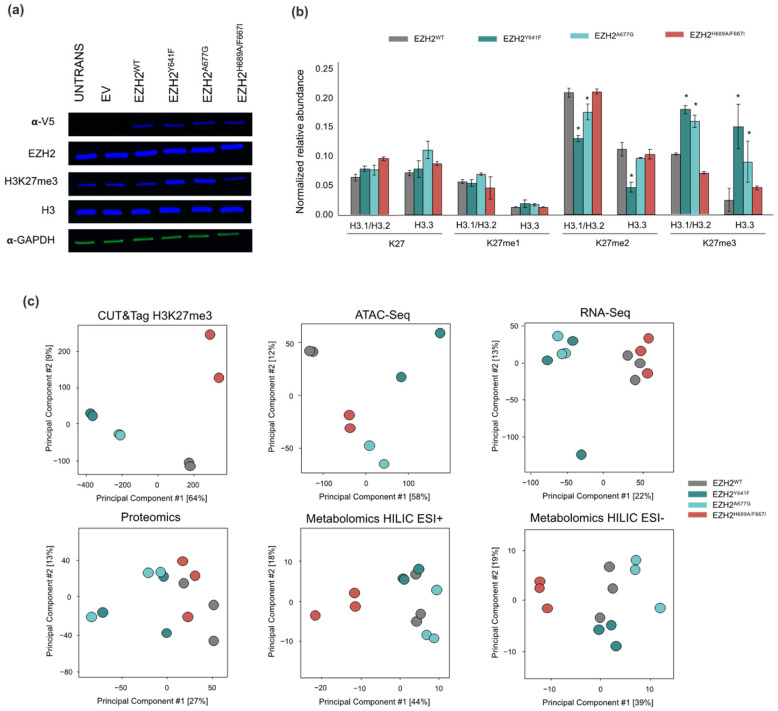
(**a**) Western blot of EZH2, V-5 tag, H3K27me3, and H3 total for untransfected, empty vector (EV), wild-type (WT) EZH2, EZH2^Y641F^, EZH2^A677G^, and EZH2^H689A/F667I^ with α-GAPDH as loading control. (**b**) Summary of EpiProfile analysis of K27 methylation for histone H3 variants across EZH2 mutants. Error bars represent 1 standard deviation and asterisk represents statistical significance (q-value < 0.05). (**c**) Principal component analysis on replicates of acquired multi-omics datasets.

To study the effects of EZH2 point mutations in H3K27me3 deposition, chromatin accessibility and downstream effects on transcription, we generated contrasting EZH2 mutants with GOF (EZH2^Y641F^ and EZH2^A677G^) and LOF (EZH2^F667I/H689A^) methyltransferase activity. H3K27me3 levels were determined for all the EZH2 mutants and controls by immunoblotting (Figure 1a) and EpiProfile mass spectrometry analysis (Figure 1b) to confirm the enrichment/depletion of K27me3 on histone H3 variants. The EZH2 GOF mutants showed increased levels of H3.1/H3.2/H3.3K27me3 concomitant with depletion of H3.1/H3.2/H3.3K27me2 levels. The H3K27me3 to H3K27me2 ratio was higher in EZH2^Y641F^ mutant. This epigenetic shift in H3K27me3 is consistent with the reported phenotype with EZH2 heterozygous mutations, where the EZH2^WT^ preferentially mono-methylates unmodified H3K27 and primes H3 for the EZH2 GOF mutants that efficiently methylate H3K27me2 due to its higher affinity and enhanced activity towards di-methylated substrates, resulting is an overall shift in the H3K27me3/H3K27me2 ratio [40,41]. In contrast, the EZH2 LOF mutant displayed lower levels of H3.1/3.2/H3.3K27me3, and an enrichment of the unmodified H3.1/H.2/H3.3K27. Interestingly, associated H3.3K36me2 levels were correlated with H3K27me3 in that the EZH2 GOF mutants showed decreased H3K36me2 whereas the LOF mutant showed an increase (Supplementary Figure S1). H3K36me2 is a well-known epigenetic mark associated with active genes and PRC2 inhibition that causes a mutually exclusive colocalization with H3K27me3 mediated by NSD1 [42,43,44].

Different molecular analyses were used to examine the altered dynamics of molecular networks driven by EZH2 mutations. A multi-omic data-driven analysis of cellular processes upset by EZH2 mutation was carried out from the experimental data obtained by H3K27me3 CUT&Tag, ATAC-Seq, RNA-Seq, label-free proteomics and untargeted metabolomics on isogenic EZH2 GOF and LOF mutants. Biological replicates for each set of experiments were grouped as shown in the principal component analysis (PCA) with unsupervised clustering of replicates across all omics platforms (Figure 1c). Moreover, the EZH2 GOF mutants were fairly discriminated by the principal component 1 (PC1) in all the datasets. Interestingly, the variability in the different omic datasets appears to increase moving from chromatin structure to gene expression to protein abundance. Supervised clustering was also performed using Data Integration Analysis for Biomarker discovery using Latent variable approaches for Omics studies (DIABLO [45]) to integrate datasets that established a subset of variables that were able to discriminate between the treatment groups (Supplementary Figure S2).

### 2.2. Characterization of Putative EZH2 Targets

**Figure 2:**
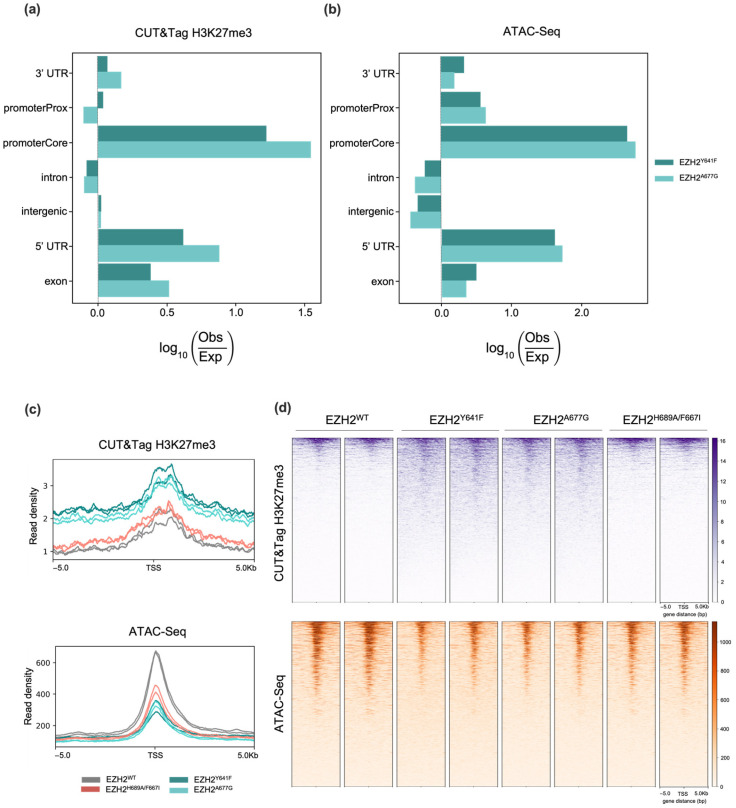
Normalized genomic distributions of (**a**) H3K27me3 CUT&Tag enriched peaks; and (**b**) Open chromatin regions in EZH2 GOF mutants. Profile plot (**c**) and heatmaps (**d**) of CUT&Tag H3K27me3 and ATAC-Seq peaks ± 5 kb from the centers of putative EZH2 targets across EZH2 mutants.

Genome-wide H3K27me3 deposition patterns in EZH2 mutants were characterized via CUT&Tag. The differential analysis confirmed an overall enrichment of H3K27me3 in EZH2 GOFs located in promoter genomic regions (Figure 2a), corroborating H3K27me3 preferential deposition around the transcription start sites (TSSs) [46]. Nearly 50% of those H3K27me3 CUT&Tag enriched peaks overlapped with H3K27me3 Chip-seq enriched regions from DLBCL cells with heterozygous Y641N (KARPAS-422), Y641F (WSU-DLCL2) or A677G (Pfeiffer) EZH2 mutations from McCabe et al. [47] (Supplementary Figure S3). Moreover, an ATAC-Seq assay was performed to identify differential open chromatin regions (OCR) and the potential binding of transcription factors (TFs). Significant depleted OCRs were predominant at promoter genomic regions (Figure 2b). Several binding sequence motifs were enriched in differential OCRs of EZH2 GOF mutants, including GATA4, HOXB9, SMAD4, and ZIC2, suggesting changes in H3K27me3 affect the binding of transcription factors (Supplementary Table S1). Figure 2a,b exhibit a similar profile in genomic distributions for both EZH2 GOF mutants in the CUT&Tag and ATAC-Seq differential regions, respectively. Additionally, similarity in the CUT&Tag and ATAC-Seq major genomic features was observed, including promoter Core, 3′ UTR, 5′ UTR, and exon regions. These observations are consistent with technical and biological reproducibility.

**Figure 3:**
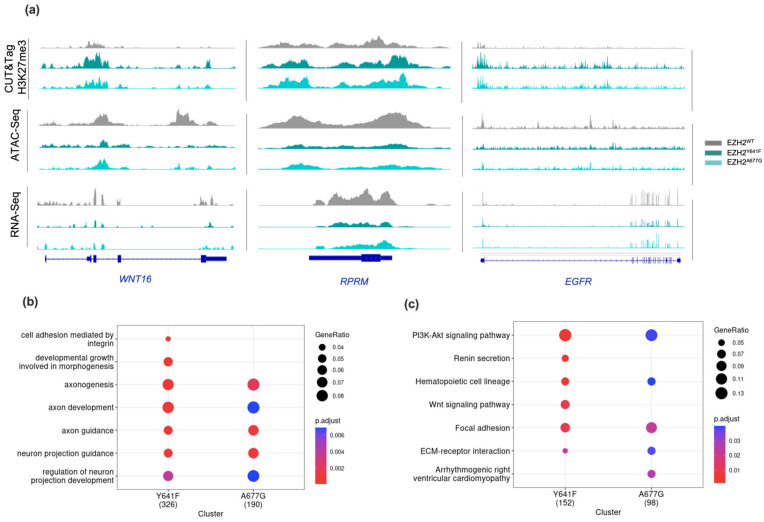
(**a**) Gene browser tracks showing CUT&Tag H3K27me3, ATAC-Seq, and RNA-Seq readouts over *WNT16*, *RPRM* and *EGFR* genes. (**b**) Gene ontology and (**c**) KEGG pathways of EZH2 target genes in EZH2 GOF mutants.

Correlation of H3K27me3 CUT&Tag peaks with differential OCRs from ATAC-Seq analysis accounted for 5269 (EZH2^Y641F^) and 1395 (EZH2^A667G^) genes showing simultaneous H3K27me3 enrichment and low chromatin accessibility, consistent with EZH2 PRC2-dependent targeting (Figure 3a). A total of 1299 putative EZH2 targets were observed across both EZH2 GOF mutants, which clustered in several genomic regions, including chromosomes Chr3, Chr6, Chr13 and Chr18 (Supplementary Figure S4). No genes were common between CUT&Tag and ATAC-Seq datasets for EZH2^F667I/H689A^. In fact, the EZH2 LOF mutant epigenetic landscape remains close to the WT, suggesting compensation by the enhancer of zeste homolog 1 (EZH1) to maintain H3K27me3 levels as observed in other EZH2 loss-of-function studies [48,49,50].

Overall gene expression was assessed through RNA-Seq analysis. Accordant with the transcriptional repressive role of H3K27me3, EZH2 GOF mutants showed a higher number of downregulated transcripts in comparison to the EZH2 LOF (Supplementary Figure S5). Gene expression profiles of EZH2 GOF were in accordance with dysregulated genes reported in different EZH2 silencing studies [51,52,53]. A total of 360 genes for EZH2^Y641F^ and 209 for EZH2^A677G^ were characterized by showing a simultaneous downregulation (q-value < 0.05) of transcript levels and chromatin accessibility, along with a significant enrichment (q-value < 0.05) of H3K27me3 (Supplementary Figures S6 and S7). Common EZH2 targets in both EZH2 GOF mutants accounted for a total of 108 genes (Supplementary Table S2). These include the gene transcripts for *COL4A4*, *EGFR*, *RPRM* and *WNT16* (Figure 3a) which have been shown previously to be regulated by EZH2:PRC2 [51,54,55,56,57]. The differential expression on those targets and the EZH2 regulator *MALAT1* were further verified using qPCR, validating that its transcript expression in GOF mutants was significantly lower compared to EZH2^WT^ (Supplementary Figure S8). We observed the transcriptional downregulation of other genes such as *ACADL*, *CD1D*, *CD55*, and *MET* among others have also been associated with EZH2 [58,59,60,61,62,63,64,65,66,67,68]. Gene ontology (GO) annotation on the downregulated genes revealed several terms associated with cell adhesion, cell morphogenesis, and cell projection organization (Figure 3b). Biological processes involving neural growth and maturation agree with EZH2’s role in axonal regeneration [69,70,71]. Moreover, KEGG pathway analysis was enriched of pathways associated with cancer, focal adhesion, and hematopoietic cell lineage (Figure 3c). Pathways related to cardiomyopathy were also enriched, consistent with reports of EZH2 regulation of cardiac genes [72,73,74].

In EZH2 GOF mutants, the mutation of either Y641F or A677G caused an overall increase in H3K27me3 deposition on promoter genomic regions with a consequential decrease in chromatin accessibility and transcriptional levels. The agreement between epigenetic dysregulation for the two EZH2 GOF mutants was considerable but with greater chromatin alterations for the EZH2^Y641F^ mutant. This difference has been reported in previous independent studies and it has been suggested to be linked to a higher efficacy of Y641F accommodating H3K27me2 at the intersection of the two binding pockets in the active site of EZH2 compared to A677G [75,76]. The putative EZH2 targets we observed are consistent with previously reported targets in EZH2 studies using different cell types (Supplementary Table S2), suggesting that some EZH2 targets are ubiquitous. Our data also suggest the existence of additional EZH2 targets that have not yet been previously described in the literature, including *ADORA1* involved in adenosine receptor signaling [77], the one-carbon metabolism enzyme *CA8* [78], and the developmental proteins *ENC1*, *FAT3*, and *NRP2* [79]. H3K27me3 epigenetic marking on those genes can favor abnormal growth, proliferation, and motility of cell populations with particularly high renewal rates, such as stem and progenitor cells. In fact, six of the EZH2 canonical targets (*CD1D*, *CD55*, *CR2*, and *ITGA2/4/6*) have a key role in controlling the homeostasis of the hematopoietic cell lineage pathway.

### 2.3. Protein Networks Are Impacted by EZH2 Activity

**Figure 4:**
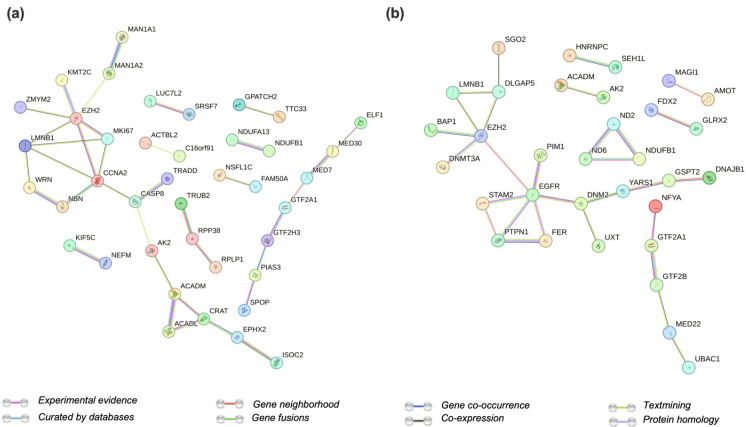
String analysis of protein–protein networks for dysregulated proteins in (**a**) EZH2 GOF mutants and (**b**) EZH2 LOF.

Label-free quantitative proteomics was performed to interrogate how changes in gene expression seen in EZH2 mutants translates to the cellular proteome. Interestingly, a higher number of significantly upregulated proteins (sixty-seven proteins) was found in both EZH2 GOF mutants (Supplementary Tables S2 and S3) associated with the following biological processes: carnitine metabolic process, mitotic cell cycle, and positive regulation of transcription. Several transcription factors (CC2D1A, FOXK2, TEAD1) and transcriptional regulators (GTF2A1, GTF2H3, PIAS3, PRNP, TRADD, WRN, ZMYM2) were found dysregulated in both EZH2 GOF mutants. STRING [80] analysis identified a network of protein interactions with EZH2 (Figure 4a). These included the known interactions with Cyclin-A2 (CCNA2), histone methyltransferase KMT2C, and proliferation marker protein Ki-67 (MKI67). There were 15 common downregulated proteins for both EZH2 GOF mutants (Supplementary Tables S2 and S3). For the EZH2 LOF mutant, the dysregulated proteins were related to gene ontology terms for mitochondrial electron transport, NADH to ubiquinone, and negative regulation of transcription. Transcription factors (GTF2B, NR2C1, NKX2–4, ZNF579, ZNF706) along with regulators (FER, GTF2A1, PIM1, and SCOC) were also found significantly altered (Supplementary Tables S3 and S4). Furthermore, the DNA methylase DNMT3A, and the epidermal growth factor receptor EGFR were present in the network of EZH2 interactors for the EZH2 LOF mutant (Figure 4b and Supplementary Table S5). Classical gene enrichment analysis was also performed on the differentially expressed proteins for all pair-wise comparisons. However, gene ontology terms and pathways show low enrichment. Moreover, considering that proteomics analysis revealed a higher proportion of upregulated proteins in the EZH2 GOF mutants (opposed to RNA-Seq), we speculate that differences are more closely related to changes in protein interaction network than to transcriptional repression.

The observed upregulation in the EZH2-interacting proteins Cyclin A2 and Ki-67 indicate a higher proliferation state associated with EZH2 GOF mutants. This is supported by the general increase in observed protein abundance (q-value > 0.05) for several transcription factors and transcriptional regulators promoting transcription as described above. Moreover, KMT2C, also called myeloid/lymphoid or mixed-lineage leukemia protein 3 (MLL3), may act as a tumor suppressor in an effort to restore homeostasis given its methyl-transferase activity towards H3K4 [81].

The combined transcriptomics and proteomics datasets with q-value < 0.05 for the EZH2^Y641F^ mutant showed an overlap in: (1) the downregulated transcriptional (BASP1, MAGED2) and translational (TCOF1) regulators; (2) the upregulated kinases (CLK4, PTK2), along with C16orf87, GPC3, FKBP9, EXOC4, MSL2, NCOA2, VCPIP1, SH3BGRL, SELENOF, and ZMYM2. For EZH2^A677G^, proteins/transcripts pairs included (1) the downregulated adenylate kinase AK2 and FAM107B; (2) the upregulated proteins involved in mitotic cytokinesis (RASA1, SEPTIN6) and response to oxidative stress (SLC23A2, WRN), together with FRYL, NDUFA13, TUBA1A, and ZNF711. For the EZH2 LOF mutant, only the upregulation of the tyrosine kinase FER was observed in both the RNA-Seq and proteomics datasets.

The comparison of proteomic data with ATAC-Seq and transcriptomics (q-value < 0.05) offered a valuable approach to identifying and validating molecular networks. Upregulation of PRNP, along with CA2 and NEFM downregulation was found in both GOF mutants overlapping with the observations from the RNA-Seq and proteomics datasets. Both CA2 and NEFM have been found upregulated in EZH2 knockout cell lines, suggesting these gene products are PRC2 targets [7,82]. Furthermore, for EZH2^Y641F^ the transcription factors ATF1 and HOXB9 were found upregulated in the proteomics analysis and its corresponding binding motif was significantly enriched. Additionally, upregulated PIAS3 interacts with transcription factors SMAD3 [83] and RELA [84], which binding motifs are also enriched in EZH2^A677G^.

### 2.4. EZH2 Mutants Alter the Celullar Metabolome

**Figure 5:**
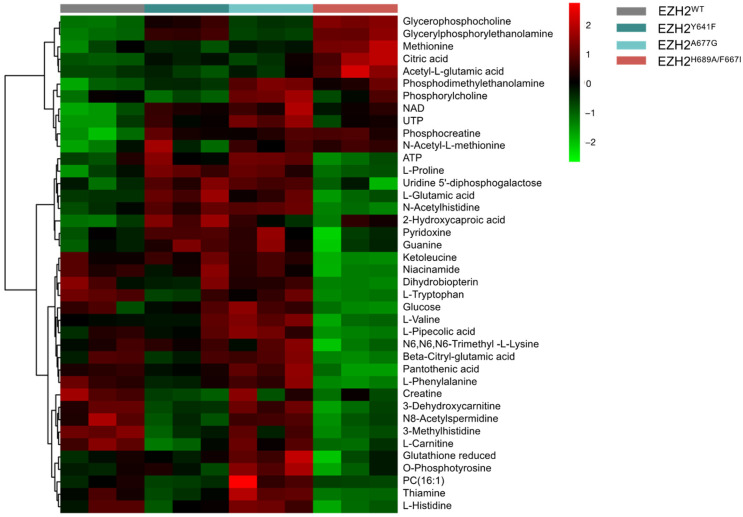
Heatmap showing top 40 differential metabolites across each EZH2 mutant.

Metabolic signatures were explored using an untargeted MS-based metabolomics analysis. Figure 5 shows the top 40 differential metabolites across each mutant. Both EZH2 GOF mutants showed an upregulation of common metabolites, including ATP, guanine, pyridoxine, L-proline, L-glutamate, and N-acetylhistine. High levels of reduced glutathione, phosphotyrosine, and UTP were found in EZH2^A677G^, while EZH2^Y641F^ showed high levels of 2-Hydroxycaproic acid. The EZH2 LOF mutant presented a significant upregulation of acetyl-L-glutamate, citrate, and L-methionine, along with downregulation of niacinamide, trimethyl-lysine, and ketoleucine.

Effects of EZH2 mutations were also explored by integrating metabolic and genomic pathways using MetaboAnalyst 5.0 [85] to identify significant molecular interacting networks (Supplementary Figure S9). EZH2 GOF mutants showed an impact in mucin type O-glycan biosynthesis, nicotinate and nicotinamide metabolism and TCA cycle pathways. For the EZH2 LOF mutant, arginine/proline metabolism, glycolysis/gluconeogenesis, and glycerophospholipid metabolism were the most altered pathways.

**Figure 6:**
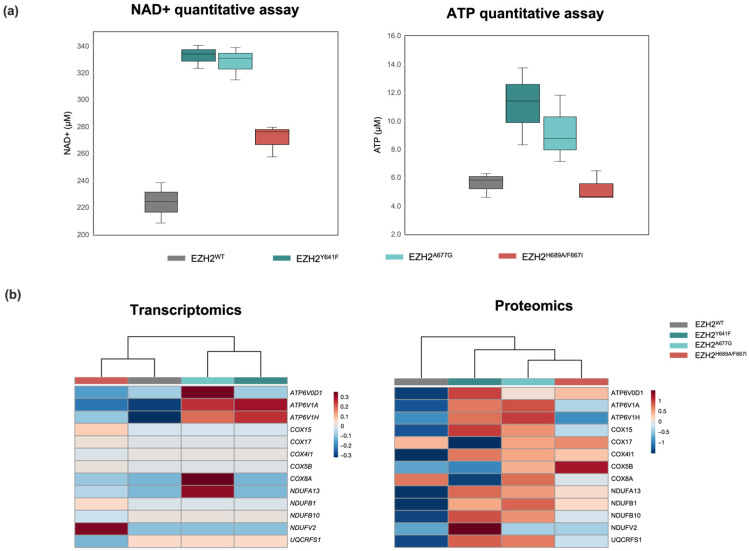
(**a**) Boxplots for NAD+ and ATP quantification via colorimetric assay and (**b**) expression heatmap for selected genes belonging to NAD+/NADH biochemical pathway in EZH2 mutants.

Dysregulation of NAD+ and ATP across mutants was validated via colorimetric assay (Figure 6a). These changes reflect alterations in the mitochondrial electron chain to increase ATP production. In fact, several subunits of the NADH dehydrogenase complex, in charge of NADH to NAD+ conversion, were found upregulated in the proteomic analysis, including NDUFB1, NDUFV2, and NDUFA13 (Figure 6b). The enzymes NAMPT and SIRT5 involved in the NAD+ salvage pathway also showed overexpression in EZH2 GOF mutants. The increase in cellular NAD+ pools is a hallmark of cancer stem cells that have been characterized in acute myeloid leukemia (AML) [86] and DLBCL [87]. Moreover, studies have shown that ATP production is impaired when EZH2 activity is inhibited [88].

In EZH2 GOF, higher levels of ATP, UTP, and glutamate can fuel cell growth and proliferation [23,89]. Moreover, both NAD+ and glutathione are crucial metabolites to maintain cellular redox homeostasis. Considering that trimethyl-lysine can only be produced from the degradation of proteins with trimethylated lysine residues, its downregulation in the EZH2 LOF mutant reflects a decrease in overall methyltransferase activity [90]. Furthermore, accumulation of the essential amino acid methionine, required for the biosynthesis of the methyl donor SAM, suggests a higher requirement to supply the activity of methyl-transferases for chromatin remodeling [91]. Higher availability of citrate can be the product of higher de novo fatty acid synthesis, in fact, inhibition of EZH2 increases the expression of FA synthesis genes [92].

## 3. Materials and Methods

### 3.1. Cell Lines and Culture

HEK-293T cell line [American Tissue Culture Collection (ATCC), CRL-11268] was cultured and maintained according to the supplier’s protocols. Cells from cryopreservation and passages were routinely tested for mycoplasma contamination using the MycoSensor PCR Assay Kit (Agilent, 302108, Santa Clara, CA, USA). HEK293T cells were chosen as a stable isogenic background model for EZH2 mutagenesis considering its early cell differentiation state; readability for stable transfection and culturing; optimal expression platform for protein production; and its non-EZH2 mutational status.

### 3.2. Constructs and Retroviruses

Stable cell line constructs carrying EZH2 GOF and LOF mutations were generated using HEK-293T cells for lentivirus production and infection. Lentivirus vectors were packaged in HEK-293T cells by transient transfection using the PEI protocol [93], containing one of the following: empty backbone pLX304 vector (control), pDEST40 vector with wild-type EZH2^WT^, gain-of-function (GOF EZH2^Y641F^ or EZH2^A677G^) or loss-of-function (LOF, EZH2^H689A/F667I^), in combination with psPax2 and pMΔ2.G. After 24 and 48 h post-transfection virus was collected and filtered using a 0.45 μM filter. Infection of HEK-293T with the generated lentivirus was carried out in presence of polybrene for 16 h in DMEM. Infected cells were selected with blasticidin (Gibco, A1113902, Billings, MT, USA) for 10 days to ensure monoclonal populations.

### 3.3. CUT&Tag

Approximately 100,000 cells were harvested with PBS at room temperature. Cells were centrifuged during 3 min at 600× *g* at RT and resuspended in 100 µL of nuclear extraction buffer (20 mM HEPES–KOH, pH 7.9, 10 mM KCl, 0.1% Triton X-100, 20% Glycerol, 0.5 mM Spermidine, 1× EDTA-free protease inhibitor) and incubated for 10 min on ice. Mixture was centrifuged at 600× *g* at 4 °C, supernatant discarded and nucleai resuspended in 100 µL of nuclear extraction buffer. Resuspended nucleai was conjugated to 10 µL activated concanavalin A-coated beads (EpiCypher, 21-1401, Durham, NC, USA) and incubated for 10 min at RT. Primary antibody incubation was performed overnight at 4 °C in 50 µL of antibody buffer (20 mM HEPES–KOH, pH 7.9, 150 mM NaCl, 0.5 mM spermidine, 0.01% digitonin, 2 mM EDTA, 1× EDTA-free protease inhibitor) with 5 µL of H3K27me3 antibody (Cell Signaling Technologies, 9733S, Danvers, MA, USA). Incubation of the secondary antibody was performed for 30 min at RT with 1:100 dilution of anti-rabbit (EpiCypher, 13-0047) in 50 µL of Digitonin150 Buffer (20 mM HEPES–KOH, pH 7.9, 150 mM NaCl, 0.5 mM spermidine, 0.01% digitonin, 1× EDTA-free protease inhibitor). Subsequently, beads bound to nuclei were mixed with 2.5 µL of 20× CUTANA pAG-Tn5 (EpiCypher, 15-1017) in 50 µL of Digitonin300 Buffer (20 mM HEPES–KOH, pH 7.9, 300 mM NaCl, 0.5 mM spermidine, 0.01% digitonin, 1× EDTA-free protease inhibitor) and incubated for 1 h at RT. The Tagmentation reaction was carried out for 1 h at 37 °C in 50 µL Tagmentation Buffer (20 mM HEPES–KOH, pH 7.9, 300 mM NaCl, 0.5 mM spermidine, 0.01% digitonin, 1× EDTA-free protease inhibitor, 10 mM MgCl_2_) and halted by removal of supernatant and wash with 50 µL of TAPS Buffer (10 mM TAPS, pH 8.5, 0.2 mM EDTA). Beads were resuspended in 5 µL of SDS release buffer (10 mM TAPS, pH 8.5, 0.1% SDS) and incubated for 1 h at 37 °C. Then, 15 μL of SDS Quench Buffer (0.67% Triton-X 100) were added and libraries were amplified using i5/i7 Nextera index primers and CUTANA High Fidelity 2× PCR Master Mix (EpiCypher, 15-1018). PCR conditions were 5 min at 58 °C, 5 min at 72 °C, 45 s at 98 °C and 17 cycles of 15 s at 98 °C—10 s at 60 °C in a Real-Time PCR System (BioRad CFX Opus 96, Hercules, CA, USA). PCR products were purified by adding 1.3× volume of Ampure XP beads (Beckman Coulter, Cat. No. A63880, Brea, CA, USA) for further elution in 15 µL of 0.1× TE buffer (Invitrogen Cat. No. 12090015, Waltham, MA, USA) and quantification using Qubit Fluorometric Quantification (Thermo Fisher, Carlsbad, CA, USA).

CUT&Tag libraries were sequenced in two independent biological replicates of each sample on Illumina NovaSeq 6000 platform by Berry Genomics Beijing Co., Ltd. (Beijing, China). Read quality was assessed with FastQC (v0.12.0) and trimmed using TrimGalore (v0.6.5). Alignment to the human genome assembly (GRCh38/hg38) was performed by BWA (v0.7.17). Mapped reads were subject to compression, sorting, and duplicate reads removal with Samtools (v1.9). MACS2 (v2.2.7.1) was used for peaks calling. CUT&Tag signal was converted to bigwig files using deepTools (v3.5.1) with normalization by Trimmed Mean of M-values (TMM). Statistical analysis was performed using DiffBind (v3.16), with q-value threshold 0.05 and 2-fold change cut-off. Visualization was carried out in the IGV genome browser (v2.16.0). Peak overlaps were established with the GenomicRanges package (v1.52.0) [94].

### 3.4. ATAC-Seq

Approximately 50,000 cells were harvested in ice-cold PBS and washed with ice-cold RSB Buffer (10 mM Tris-HCl, pH 7.4, 10 mM NaCl and 3 mM MgCl_2_). After centrifugation for 10 min at 500× *g* at 4 °C, cell pellet was resuspended in ice-cold lysis buffer (10 mM Tris-HCl, pH 7.5, 10 mM NaCl, 3 mM MgCl_2_, 0.1% NP-40) and incubated on ice for 5 min. Subsequently, DNA was purified with a MinElute PCR Purification Kit (Qiagen, Cat. No 28004, Hilden, Germany) and eluted in EB buffer. PCR amplification was carried out in 50 µL of reaction mix containing primers i5/i7 Nextera, 100× SYBR Green I (Life Technologies, S-7563, Carlsbad, CA, USA), NEBNext High-Fidelity PCR Master Mix (NEB, M0541S), PCR-grade water and 10 μL of transposed DNA. PCR conditions were 5 min at 72 °C, 30 s at 98 °C and 16 cycles of 10 s at 98 °C—30 s at 63 °C—60 s at 72 °C in a Real-Time PCR System (BioRad CFX Opus 96). PCR products were purified using AMPure beads (Beckman Coulter, Cat. No. A63880) in a 1.8× ratio and quantified with Qubit Fluorometric Quantification (Thermo Fisher, Carlsbad, CA, USA).

ATAC-Seq libraries were sequenced in two independent biological replicates from each sample on Illumina NovaSeq 6000 platform by Berry Genomics Beijing Co. Ltd. Sequencing qualities were evaluated with FastQC (v0.12.0) and then pre-processed using TrimGalore (v0.6.5). Trimmed sequences were aligned to human genome assembly (GRCh38/hg38) using BWA (v0.7.17). Mapped reads were subject to compression, sorting, and duplicate reads removal with Samtools (v1.9). Peaks were called using MACS2 (v2.2.7.1) and converted to bigwig files with deepTools (v3.5.1) with normalization by Reads Per Kilobase per Million mapped reads (RPKM). Differential occupancy analysis was performed using DiffBind (v3.16), with q-value threshold 0.05 and 2-fold change cut-off. Motif analysis was carried out with homer (v4.11). Bigwig files were visualized in the IGV genome browser (v2.16.0).

### 3.5. Analysis of Histone PTMs

About 1 × 10^7^ cells were washed three times with ice-cold PBS (supplemented with 1× protease inhibitor and 1× phosphatase inhibitor) and harvested by scrapping. Cell pellet was resuspended in 700 µL of lysis buffer 1 (50 mM Tris-HCl, pH 7.5, 20 mM NaCl, 1 mM EDTA, 1% Glycerol (*v*/*v*), 0.5% NP-40 (*v*/*v*), 0.25% Triton X-100 (*v*/*v*), 10 mM Sodium Butyrate, 1 mM DTT, and supplemented with 1× protease inhibitor and 1× phosphatase inhibitor) and incubated with rotation for 15 min at 4 °C. Nuclei were pelleted by centrifugation at 400× *g* for 5 min at 4 °C, and washed with 500 µL of lysis buffer 2 (50 mM Tris-HCl, pH 7.5, 20 mM NaCl, 1 mM EDTA, 0.5 mM EGTA, 2 mM Sodium Vanadate, 10 mM Sodium Butyrate, 1 mM DTT and supplemented with 1× protease inhibitor and 1× phosphatase inhibitor). After centrifugation at 400× *g* for 5 min at 4 °C, nuclear pellets were suspended in 250 µL of 0.4N H_2_SO_4_ and incubated for 2 h over ice with intermittent vortexing. Cellular debris was pelleted at >20,000× *g* for 15 min at 4 °C and supernatant transferred to 2 mL Eppendorf tube. After addition of 1.5 mL of ice-cold acetone, mixture was incubated overnight at −20 °C.

Histones were precipitated by 15,000× *g* centrifugation for 15 min at 4 °C and then washed twice with ice-cold acetone. Subsequently, histones were resuspended in ammonium bicarbonate (100 mM) and quantified using Bradford assay. Histone extract (100 μg) were mixed with acetonitrile:propionic anhydride (3:1) at pH 7.5, and then incubated for 15 min at room temperature until dry. This process was repeated four times. Then, histones were digested with trypsin (1:25 wt/wt) overnight at 400 rpm at 37 °C. Histone peptides were incubated again with acetonitrile:propionic anhydride (3:1) at pH 7.5 two times to convert all newly formed N-termini, dried in a vacuum concentrator and quantified via Nanodrop at 280nm (Thermo Fisher, Carlsbad, CA, USA).

Histone peptides (1200 ng) were injected into a PepMap C18 column (Thermo Fisher Scientific Cat No ES800A, 3 μm, 100 Å, 75 μm × 15 cm) coupled to a Q-Exactive Plus Hybrid Quadrupole Orbitrap mass spectrometer (Thermo Fisher Scientific). Chromatographic separation was carried out at 300 nL/min with mobile phase A (0.1% FA in water) and mobile phase B (0.1% FA in ACN) following this gradient: 0–5 min 2% B, 5–45 min 45% B, 45–52 min 55% B, 52–56 min 95% B, 56–64 min hold at 95% B and equilibrate to 2% B for 64–75 min. Mass spectra was collected in data-independent acquisition (DIA) mode with the following parameters: MS1—resolution 35 k, AGC target 3 × 10^6^, maximum fill time 200 ms over a scan range 300–1100 *m*/*z*, MS2—resolution 17.5 k, AGC target 1 × 10^6^ with a loop count of 8 and a sliding isolation window of 50 *m*/*z*. Data analysis was performed using EpiProfile (v2.0) [95].

### 3.6. Transcriptomics

Total RNA was isolated using the Total RNA Purification Plus Kit (Norgen Biotech, Thorold, ON, Canada) according to manufacturer’s protocol. RNA concentration and quality was assessed via Qubit and Bioanalyzer (OSU Genomics Shared Resource) prior to sequencing. RNA libraries were prepared using TruSeq Stranded mRNA Library Prep Kit (Illumina, 20020594, San Diego, CA, USA) with quality and concentration assessed prior sequencing in Illumina HiSeq 4000 platform by Berry Genomics Beijing Co. Ltd. Quality of reads was evaluated using FastQC (v0.12.0) and adapter sequences were trimmed with TrimGalore (v0.6.5). Resulting FastQ files were aligned to human genome assembly (GRCh38/hg38) using STAR (v.2.7.9a). Mapped reads were compressed and sorted with Samtools (v1.9). Transcript abundance was estimated with salmon (v.1.2.0) using Gencode v23 transcriptome as reference. Raw read counts were trimmed mean normalized and used for differential gene expression analysis with edgeR (v3.16). Differential expression was determined by a modified exact test using a q-value threshold of <0.05. Bigwig files conversion was carried out with deepTools (v3.5.1) for visualization in the IGV genome browser (v2.16.0). Gene ontology and pathway analysis were performed with the clusterProfiler package (v4.8.1) [96].

### 3.7. Proteomics

Label-free proteome analysis was performed using an average 10 million cells per sample. Cells were washed and harvested in ice-cold PBS supplemented with a complete protease inhibitor cocktail (Roche, 11697498001, Basel, Switzerland) and phosphatase inhibitor (Roche, 04906845001). Cell pellets were resuspended in RIPA Lysis and Extraction Buffer (Thermo Fisher Scientific, 89900) supplemented with protease and phosphatase inhibitors, and sonicated for 10 min at 4 °C (30 s on/30 off interval, Bioruptor, Diagenode, Seraing, Belgium). Protein concentration was assessed using the Pierce™ BCA Protein Assay Kit (Thermo Fisher Scientific, 23227).

Proteins were reduced with dithiothreitol (Thermo Scientific, A39255) for 45 min at 50 °C and alkylated with iodoacetamide (Sigma-Aldrich, A3221, St. Louis, MO, USA) for 30 min in the dark at room temperature. Phosphoric acid was added for acidification (pH 3), followed by S-Trap binding buffer addition (90% MeOH, 100 mM final TEAB, pH 7.1) and loading into an S-trap spin column. After column centrifugation, mass spectrometry grade trypsin (1:20, enzyme:substrate) (Thermo Scientific, 90058) was added and incubated overnight at 37 °C. Peptides were eluted using TEAB and were dried in a SpeedVac (Thermo Scientific, SPD130P1-115). Dried peptides were resuspended in 50 mM glacial acetic acid (Fisher Scientific, A11350) and peptide concentration assessed via Nanodrop (Thermo Scientific, 13-400-518PM3).

MS data were acquired on a Bruker timsTOF Pro HPLC–MS/MS with Ion Mobility equipped with a CaptiveSpray source (Bruker Scientific, Billerica, MA, USA) operated in positive ion mode. Peptides were loaded on a C18 column (25 cm  ×  75 μm, 1.6 μm, IonOpticks, Fitzroy, Australia). Mobile phases consisted of water with 0.1% formic acid (phase A), and acetonitrile with 0.1% formic acid (phase B) at a flow rate of 0.4 mL/min and 50 °C. Peptides were eluted with the following set up: a linear gradient from 2 to 25% mobile phase B during 90 min, 25 to 37% in 10 min, and 37 to 80% over 10 min; then kept for 10 min. The column was equilibrated in 2% of mobile phase B for 15 min before the next sample injection. Data were acquired on the Parallel Accumulation Serial Fragmentation (PASEF) acquisition mode, with a mass range of 100–1700 *m*/*z*, a capillary voltage of 1.6 kV, dry gas 3 L/min, and dry temp of 180 °C. PASEF settings included 10 MS/MS scans at 1.18-s total cycle time, scheduling target intensity of 20,000, active exclusion release after 0.4 min, and CID collision energy of 42 eV.

Raw data were converted to mzML format using ProteoWizard (v3.0.2) [97]. OpenMS (v2.6.0) [98] was employed for peptide identification with MSGF+ search engine against a reviewed UniProt human proteome FASTA file with the following parameters: full trypsin digest; enzyme, trypsin; maximum missed cleavage sites, 2; precursor mass tolerance, 10 ppm; fragment mass tolerance, 0.05 Da; carbamidomethyl of Cys, fixed modification; oxidation of Met, variable modification. PSM rescoring was completed with Percolator and protein inference was performed with Epifany [99] across all samples setting peptide and protein FDR to 0.05. Normalization and differential expression analysis were carried out using edgeR (v3.16) with a q-value threshold of 0.05. STRING (v11.5) [80] was employed for protein–protein network analysis. Gene ontology and pathway analysis were established with the clusterProfiler package (v4.8.1) [96].

### 3.8. Metabolomics

Approximately 10 million cells per sample were washed with PBS at room temperature and harvested with ice-cold methanol:water (80:20). Cell lysis was performed by consecutive freeze-and-thaw cycles with liquid N_2_/dry-ice with vortexing between cycles. Supernatant was then dried on SpeedVac concentrator (Thermo Scientific, SPD130P1–115) for storage at −80 °C. Dried extracts were resuspended in Acetonitrile:Water (25:75) with addition of 0.2 µM L-methionine-methyl-d3 (98 atom %D, ISOTEC) as the internal standard.

UHPLC–MS/MS analyses were carried out on a 1290 series chromatographic system (Agilent Technologies, Palo Alto, CA, USA) coupled to a QTOF–MS 6545 series (Agilent Technologies, Palo Alto, CA, USA) equipped with an electrospray ion source (ESI). 5 µL of resuspended extracts were injected into a Kinetex HILIC column (100 × 2.1 mm, 2.6 µm, Phenomenex). Mobile phases consisted of water with 0.1% formic acid and 10 mM ammonium formate (phase A), and acetonitrile with 0.1% formic acid and 10 mM ammonium formate (phase B) at a flow rate of 0.3 mL/min and 30 °C. Gradient elution was programmed by the following set up: 98–55% B in 45 min; 55% B during 4 min; 55–98% in 2 min; and column re-equilibration for 15 min using the initial solvent composition. Data were acquired in both, positive and negative ESI mode with a mass range from 100 to 1200 *m*/*z*, full scan mode at a scan rate of 2 scans per second, 3000 V of capillary, 10 L/min of nebulizer gas flow and 300 °C of gas temperature. For MS/MS data collection, a voltage between 10 and 30 V was applied on the selected precursor ions. Mass correction throughout the analysis was achieved by continuous pumping and monitoring of the ions *m*/*z* 121.0509 (C_5_H_4_N_4_) and 922.0098 (C_18_H_18_O_6_N_3_P_3_F_24_) for ESI+ mode, and *m*/*z* 112.9856 (C_2_F_3_O_2_(NH_4_)) and 1033.9881 (C_18_H_18_O_6_N_3_P_3_F_24_) for ESI-mode.

Raw data were converted to mzML format using ProteoWizard (v3.0.2). OpenMS (v2.6.0) was employed for feature detection, alignment, assembly, and deconvolution. Three complementary approaches were integrated for feature annotation: (1) accurate mass search, matching *m*/*z* with compounds in the Human Metabolome database (HMDB v5.0) [100], Kyoto Encyclopedia of Genes and Genomes (KEGG) and Chemical Entities of Biological Interest (ChEBI); (2) spectral search, matching MS/MS fragmentation with mass spectral records in the Global Natural Product Social Molecular Networking (GNPS) and MassBank of North America (MoNA); (3) de novo molecular structure identification, using SIRIUS (v4.8.2) [101] for structure prediction based on isotope distribution and fragmentation pattern analysis. Normalization, statistical analysis and pathway analysis were performed in MetaboAnalyst 5.0 [85] with a q-value threshold of 0.05.

### 3.9. Immunoblotting

Cells were collected and lysed in RIPA buffer (Thermo Scientific, 89900) freshly supplemented with a complete protease inhibitor cocktail (Roche, 11697498001) and phosphatase inhibitor (Roche, 04906845001). Protein concentration of the cell lysate was measured by Bradford assay (BioRad, 5000121). Equal amounts of protein lysates were separated in precast protein gels (Bio-Rad, 4568084, 4568044) and transferred to nitrocellulose blotting membrane (Biorad, 1620115) via standard wet transfer at 4 °C overnight. The membrane was blocked with TBS Blocking Buffer (Li-Cor, 927-60001, Lincoln, NE, USA) for an hour at room temperature and then incubated overnight at 4 °C with primary antibodies against EZH2 (Cell Signaling Technology, 5246), H3 (Abcam, ab1791, Cambridge, UK), H3K27me3 (Cell Signaling Technology, 9733), GAPDH (Cell Signaling Technology, 2118S) and V5 (Invitrogen, R960-25). Detection was carried out using species appropriate secondary antibodies (Li-Cor, 926-68073, 926-32212) and membrane developing with Li-Cor Odyssey Fc Imaging System. Intensity of protein bands was quantified using ImageJ (v. 1.54d) and normalized to loading control band.

### 3.10. RNA Isolation and RT-qPCR

Total RNA was isolated using the PureLink™ RNA Mini Kit (Invitrogen, 12183025). Reverse transcription was performed with 2 μg of total RNA using the High-Capacity cDNA Reverse Transcription Kit (Thermo Fisher, 4368814). qRT-PCR assays were carried out in quintuplicates using the Real-Time PCR System (BioRad CFX Opus 96) and the following TaqMan probes: *COL4A1* (Hs00266237, PN4453320), *GAPDH* (Hs99999905, PN 4453320), *EGFR* (Hs01076090, PN 4453320), *MALAT1* (Hs01910177, PN 4448892), *RPRM* (Hs04189060, PN 4448892) and *WNT16* (Hs00365138, PN 4453320) (Thermo Fisher Scientific, Carlsbad, CA, USA). Data were normalized with GAPDH as reference gene. Relative expression was calculated using the 2ΔΔCt method [102].

### 3.11. Colorimetric Assays

Cellular NAD+ was measured using a Colorimetric Assay Kit (Abcam, Cat. Ab65348) following the manufacturer’s protocol. Briefly, 1 × 10^6^ cells were harvested and washed with PBS prior lysis. Subsequently, lysates were centrifuged, and supernatants were recovered. 50 µL of supernatant were incubated with 100 µL of reaction mix for 2 h at room temperature. Absorbance was measured on a microplate reader (Molecular Devices, San Jose, CA, USA, SpectraMax iD5) at 460 nm. All experiments were performed in triplicates.

Cellular levels of ATP were measured employing an ATP Determination Kit (Invitrogen, Cat. A22066) following the manufacturer’s instructions. Approximately 1 × 10^6^ cells were harvested and washed with PBS prior lysis. Lysates were centrifuged and supernatants were recovered. A volume of 10 µL of supernatant was mixed with 90 µL of reaction solution. Luminescence was measured on a microplate reader (Molecular Devices, SpectraMax iD5). All experiments were performed in triplicates.

## 4. Conclusions

A multi-omics approach was employed to examine how clinically relevant single-point mutations on EZH2 interact to upset complex cellular networks. The EZH2 GOF mutants, Y641F and A677G, showed a consistent set of transcriptional repressive targets that disturb several pathways in cancer, focal adhesion, and hematopoietic cell lineage. Moreover, the observed dysregulation on proteins and metabolites has the capacity to sustain aberrant cell growth, proliferation, and invasiveness. Protein network analysis also revealed specific EZH2 direct interactors, including Cyclin-A2, KMT2C and Ki-67 that can contribute to amplifying remodeling effects on the epigenetic landscape. Metabolic profiles in isogenic cells responded to changes in EZH2 activity showing shifts in key biosynthetic pathways that play a role in tumor survival. The H3K27me3 deposition and chromatin accessibility of the EZH2 LOF mutant was unperturbed. However, the decrease in EZH2 activity led to important changes in gene, protein and metabolite expressions associated with tumor survival pathways. The primary limitation of this study is the actual translation of these EZH2 mutational effects into a B-cell-specific context. The data show that the observed EZH2 targets in the HEK293T cell lines that were affected by mutation have consistency with the dysregulated genes observed in B-cell lines [47,76]. In these studies, the cell lines have varying genetic backgrounds that confound the interpretation of the data. This study lays a consistent baseline for future multi-omics analysis that can be performed using patient samples with blood malignancies that harbor varying other genetic abnormalities. Comparisons with the baseline will allow for a more systematic hypothesis-driven approach to decipher the role of EZH2 mutations. Future studies will also use this model to explore the influence of spatial chromatin structure, nuclear lamina, and EZH2 interaction with other histone modifiers and chromatin remodelers.

## Data Availability

Acquired CUT&Tag data were deposited in GEO under accession number GSE235011, ATAC-Seq under accession number GSE235010, and RNA-Seq under accession number GSE235013. Additionally, the publicly available dataset GSE40970 (H3K27me3 ChIP-seq analysis in EZH2 mutant and wild-type DLBCL cell lines) was used in this study. Proteomics datasets are accessible on PRIDE through accession number PXD042953 for histone PTM analysis and PXD042875 for label-free proteomics. Metabolomics datasets are available in Metabolomics Workbench with the study ID ST002734.

## Supplementary Materials

The following supporting information can be downloaded at: https://www.mdpi.com/article/10.3390/ijms241411378/s1.
