# Supplementary material for: Integrative Multi-Omics Analysis of Oncogenic EZH2 Mutants: From Epigenetic Reprogramming to Molecular Signatures
Source: Int J Mol Sci. 2023 Jul 12;24(14):11378. doi: 10.3390/ijms241411378 (PMC10380343; doi:10.3390/ijms241411378)
Supplement: Supplementary file 1 [file ijms-24-11378-s001.zip › Supplementary_Figures.pdf]

TABLE OF CONTENTS

Supplementary Figure S1. Results of histone PTM analysis via EpiProfile across EZH2 mutants.

Supplementary Figure S2. Supervised clustering analysis on replicates of acquired multi-omics datasets.

Supplementary Figure S3: Overlapping of H3K27me3 CUT&Tag enriched peaks with H3K27me3 Chip-seq enriched regions from DLBCL cells with Y641X and A677G mutations.

Supplementary Figure S4: Distribution over chromosomes of putative EZH2 targets with enrichment of H3K27me3 and low chromatin accessibility in EZH2 GOF mutants.

Supplementary Figure S5: Volcano plots of RNA-Seq differential expression analysis for EZH2 mutants.

Supplementary Figure S6: Quantitative comparison of genes with significantly upregulated H3K27me3/downregulated chromatin accessibility/ downregulated transcript levels in EZH2 GOF mutants.

Supplementary Figure S7: Overlap of genes with simultaneous significant H3K27me3 (+)/chromatin accessibility (-)/transcript levels (-) in EZH2 GOF mutants.

Supplementary Figure S8: Quantification of selected gene transcripts using qPCR.

Supplementary Figure S9: Pathway analysis results from MetaboAnalyst of the differential metabolic signatures in EZH2 mutants.

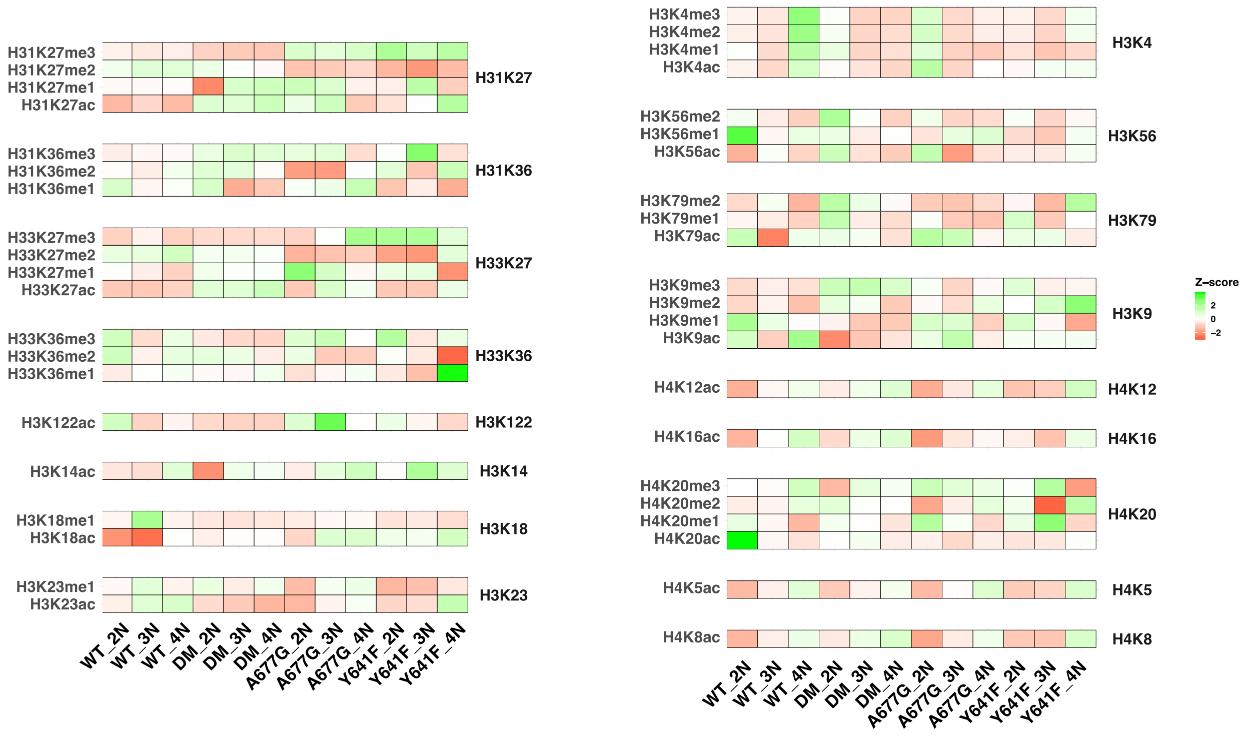

Supplementary Figure S1. Results of histone PTM analysis via EpiProfile across EZH2 mutants.

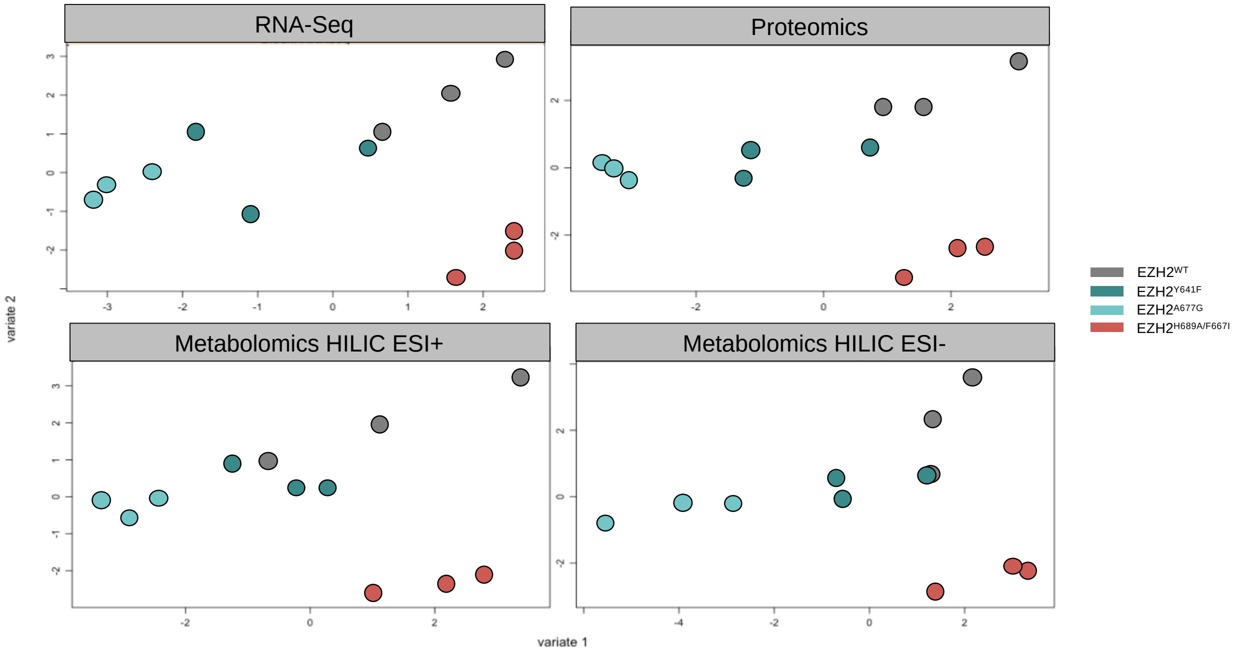

Supplementary Figure S2. Supervised clustering analysis on replicates of acquired multi-omics datasets.

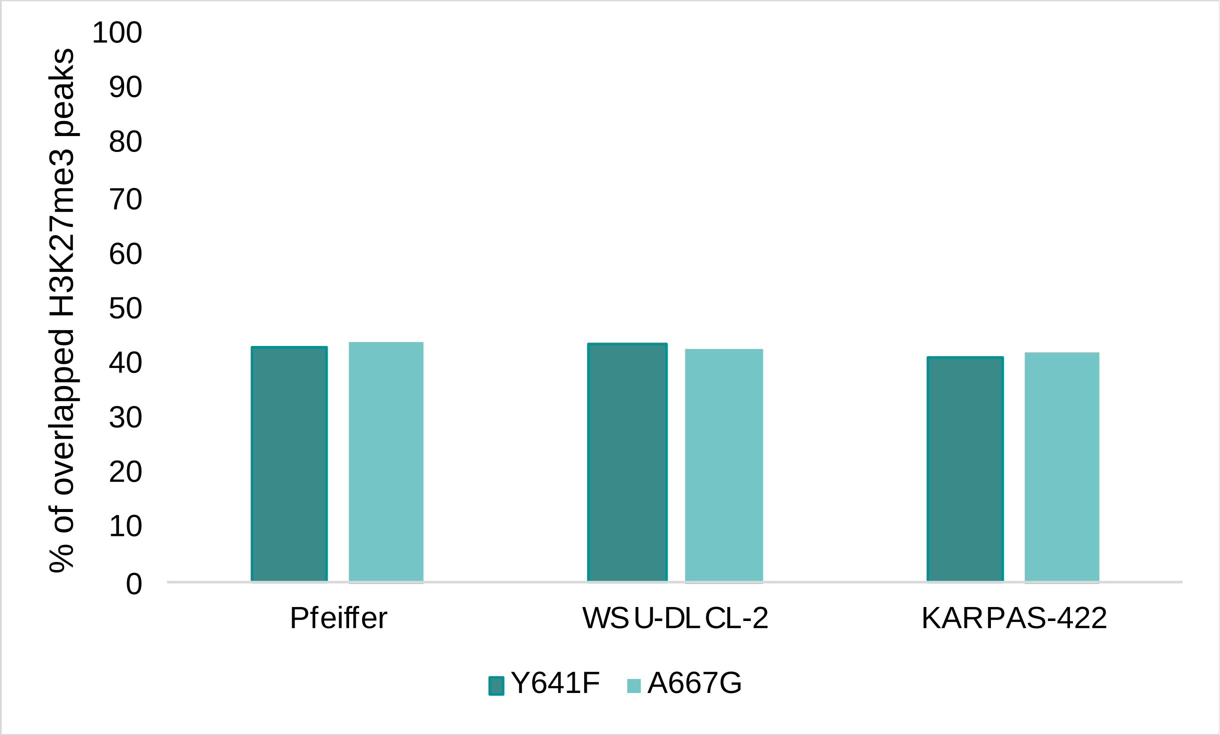

Supplementary Figure S3. Overlapping of H3K27me3 CUT&Tag enriched peaks with H3K27me3 Chip-seq enriched regions from DLBCL cells with Y641X and A677G mutations.

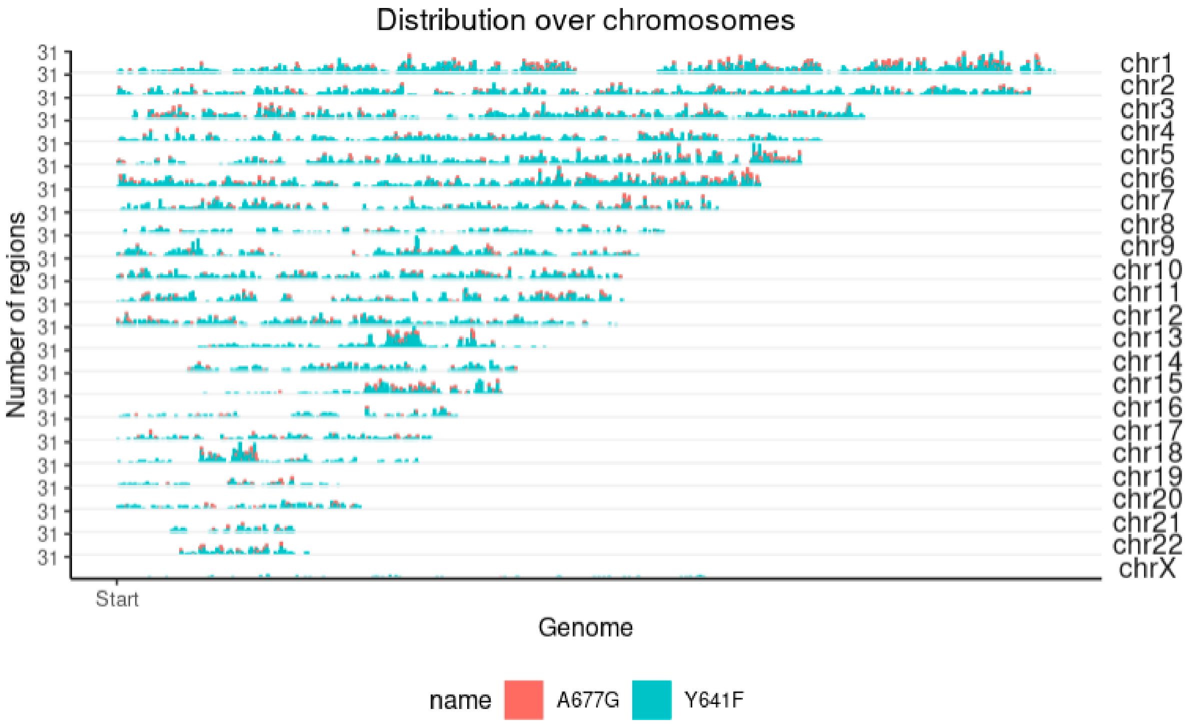

Supplementary Figure S4. Distribution over chromosomes of putative EZH2 targets with enrichment of H3K27me3 and low chromatin accessibility in EZH2 GOF mutants.

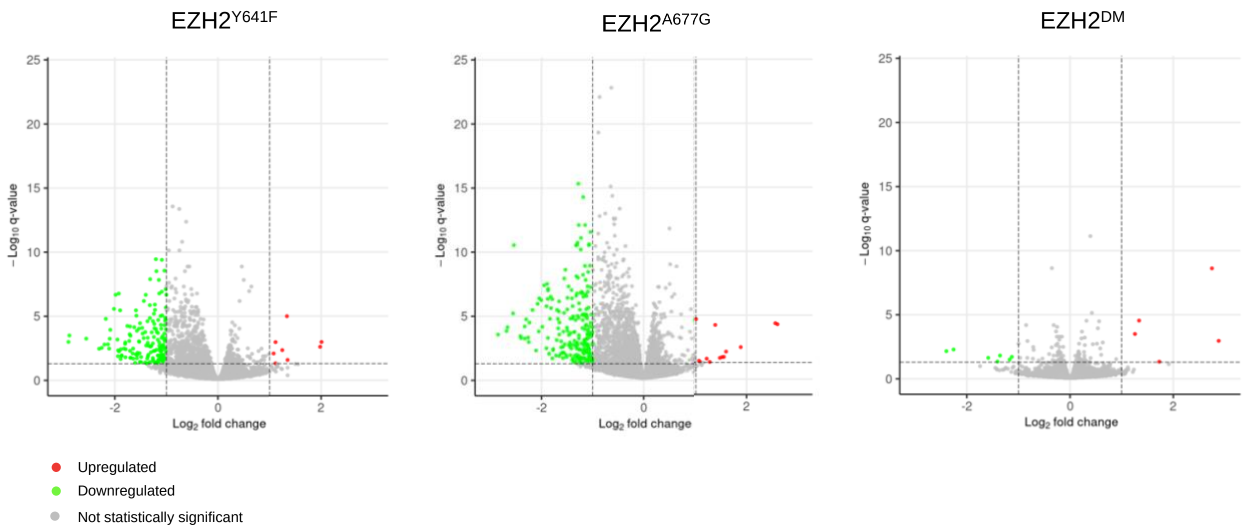

Supplementary Figure S5. Volcano plots of RNA-Seq differential expression analysis for EZH2 mutants.

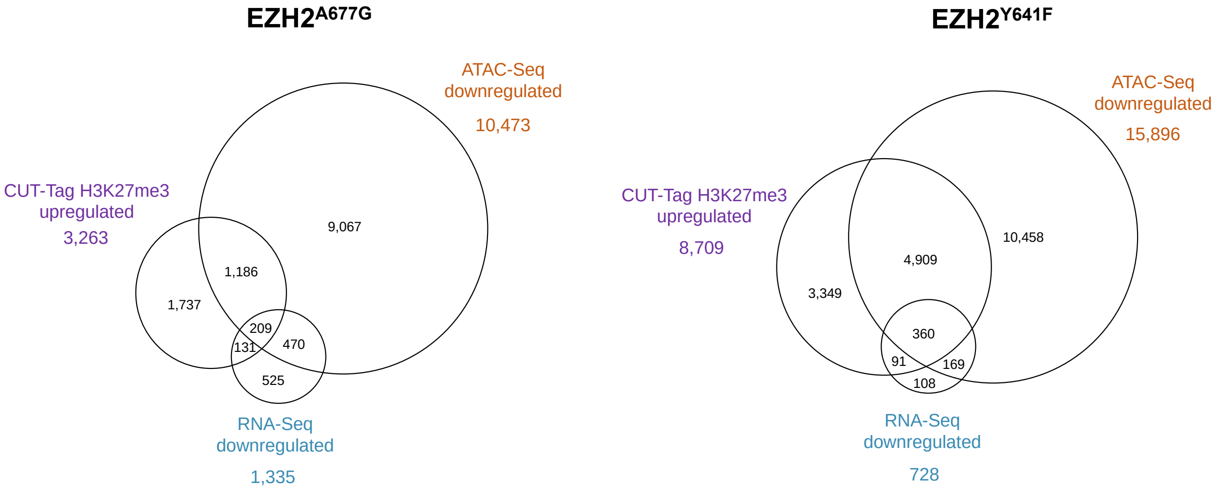

Supplementary Figure S6. Quantitative comparison of genes with significantly upregulated H3K27me3/downregulated chromatin accessibility/downregulated transcript levels in EZH2 GOF mutants.

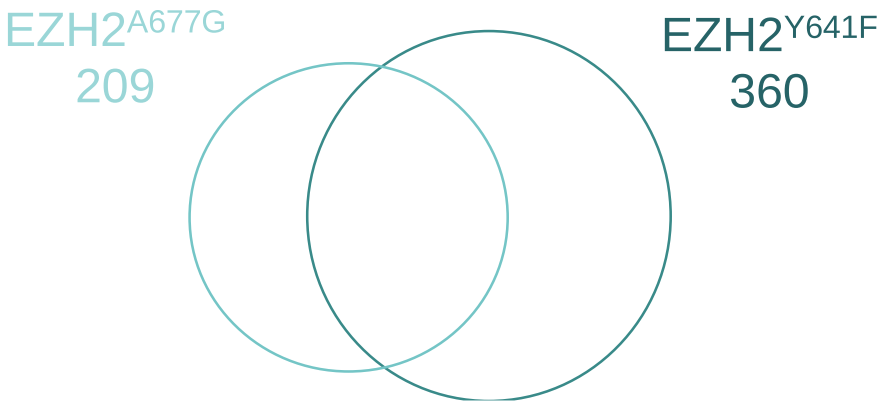

Supplementary Figure S7. Overlap of genes with simultaneous significant H3K27me3 (+)/chromatin accessibility (-)/transcript levels (-) in EZH2 GOF mutants.

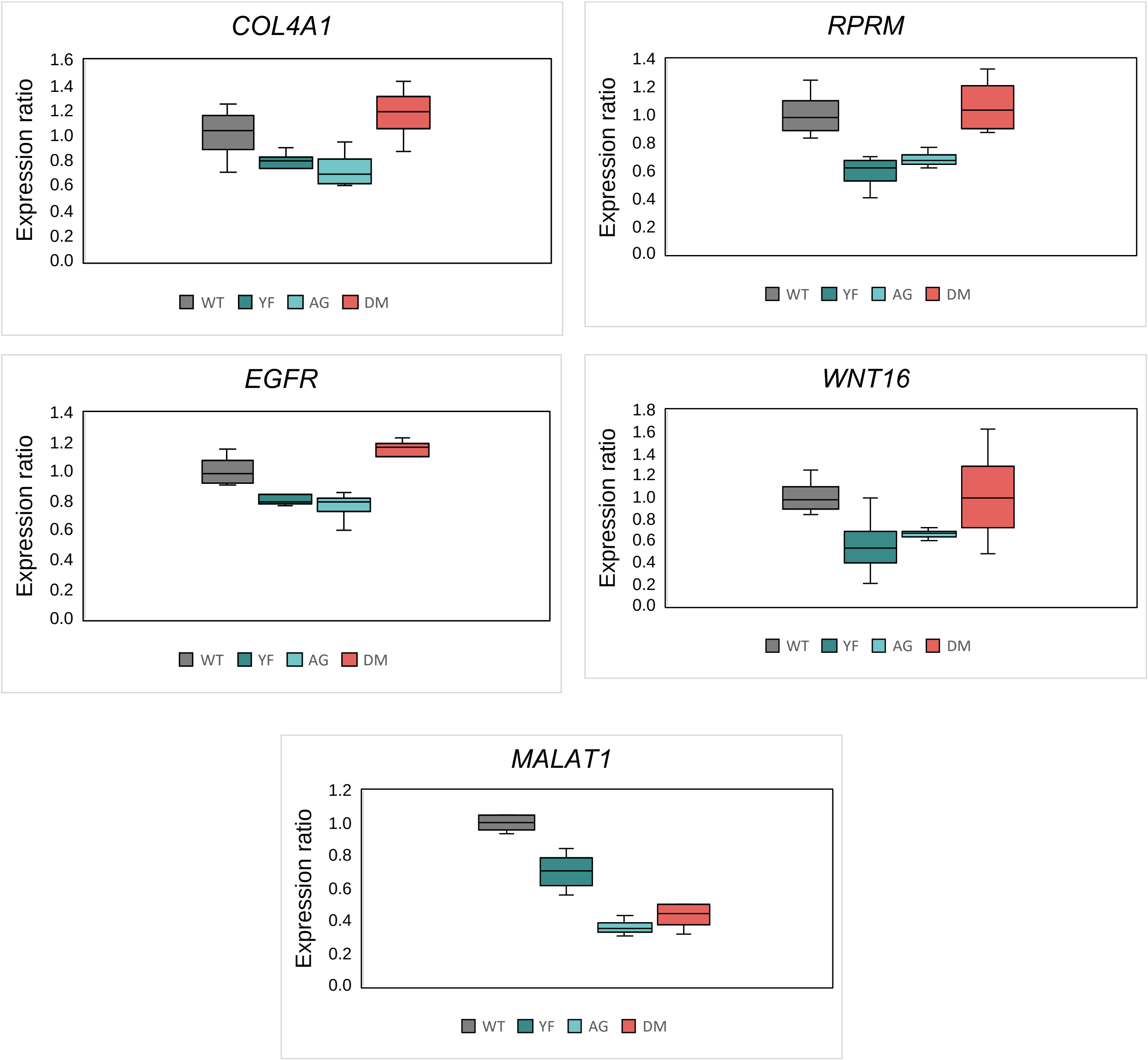

Supplementary Figure S8. Quantification of selected gene transcripts using qPCR.

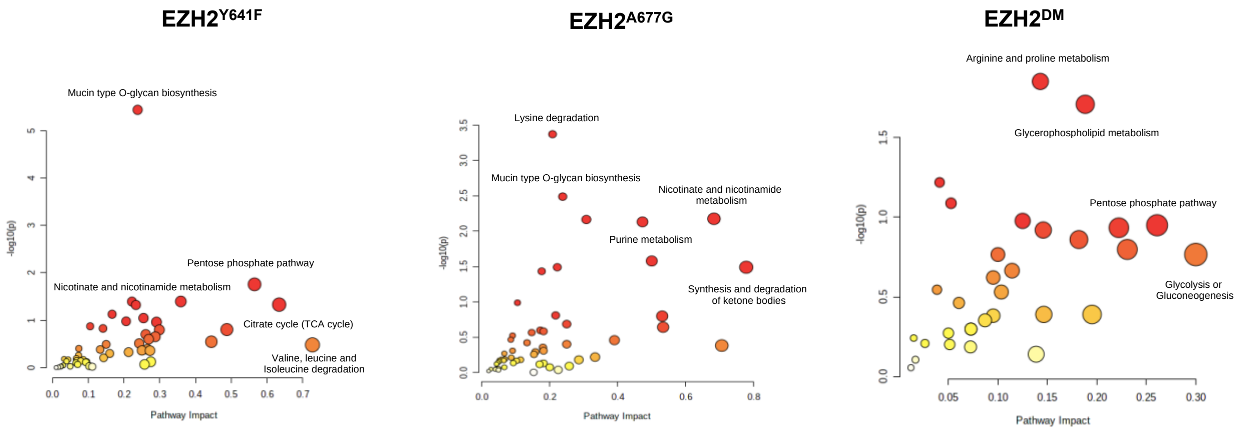

Supplementary Figure S9. Pathway analysis results from MetaboAnalyst of the differential metabolic signatures in EZH2 mutants. Fill color represents the degree of disturbance in the pathway, from white (low significance) to red (higher significance).
