# Supplementary material for: Integrative Multi-Omics Analysis of Oncogenic EZH2 Mutants: From Epigenetic Reprogramming to Molecular Signatures
Source: Int J Mol Sci. 2023 Jul 12;24(14):11378. doi: 10.3390/ijms241411378 (PMC10380343; doi:10.3390/ijms241411378)
Supplement: Supplementary file 1 [file ijms-24-11378-s001.zip › Supplementary_Table_S1.pdf]

Supplementary Table S1. ATAC-Seq enriched motifs in EZH2 GOF mutants.

Name	Composite Logo	p-value	
		EZH2^{Y641F}	EZH2^{A677G}
ATF1	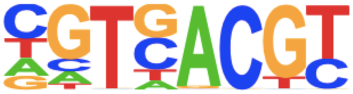	1E-169	NF
FOXF1	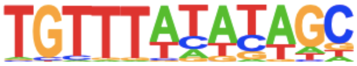	NF	1E-68
FOXO1	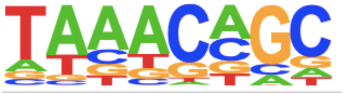	1E-249	NF
GATA4	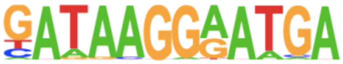	1E-64	1E-41
HOXB9	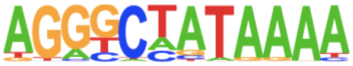	1E-53	NF
RELA	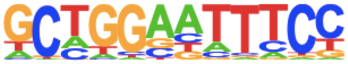	NF	1E-56
SMAD3	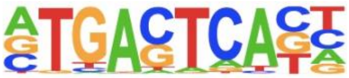	1E-129	NF
SMAD4	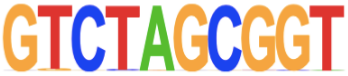	1E-86	1E-27
ZIC2	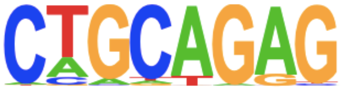	1E-164	1E-59

NF, Not found significant.
